# Supplementary material for: Valorization of Thyme Combined with Phytocannabinoids as Anti-Inflammatory Agents for Skin Diseases
Source: Pharmaceutics. 2025 Oct 2;17(10):1291. doi: 10.3390/pharmaceutics17101291 (PMC12567430; doi:10.3390/pharmaceutics17101291)
Supplement: Supplementary file 1 [file pharmaceutics-17-01291-s001.zip › pharmaceutics-3829228-supplementary.pdf]

Supplementary Materials: Valorization of Thyme Combined with Phytocannabinoids as Anti-Inflammatory Agents for Skin Diseases

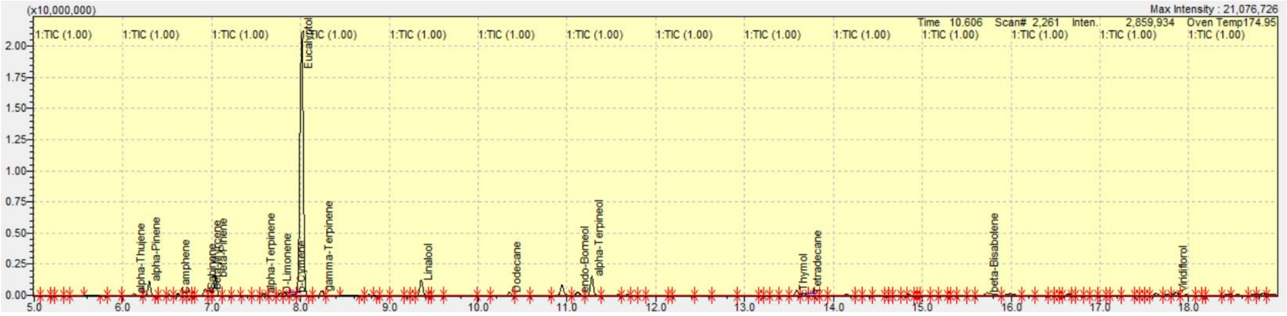

Figure S1. – GC-MS chromatogram of hydrodistillate of *Thymus mastichina* essential oil.

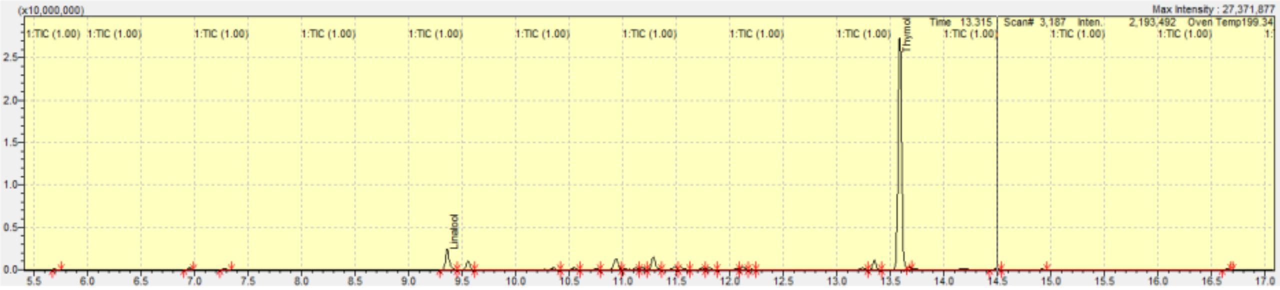

Figure S2. – GC-MS chromatogram of FA.

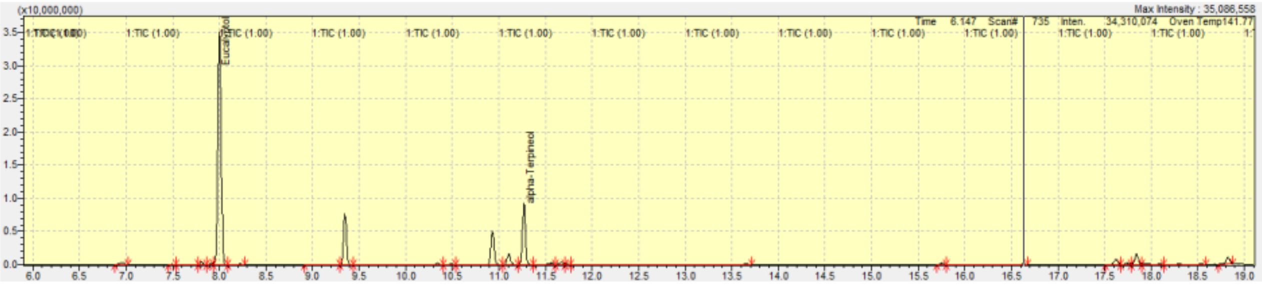

Figure S3. – GC-MS chromatogram of FB.

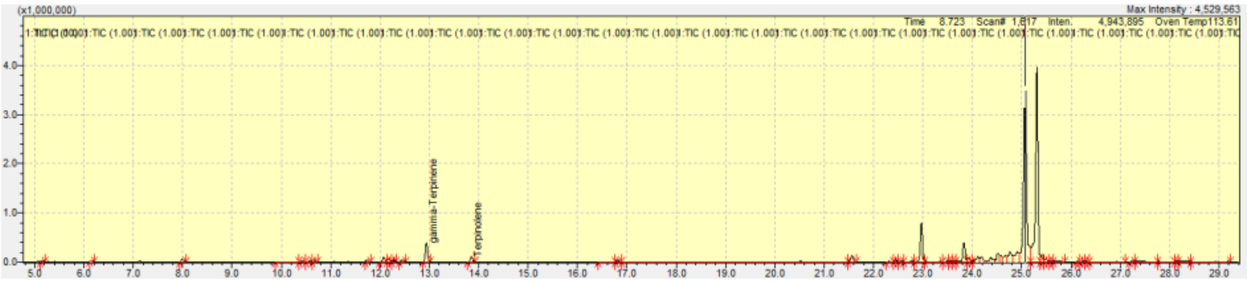

Figure S4. – GC-MS chromatogram of FC.

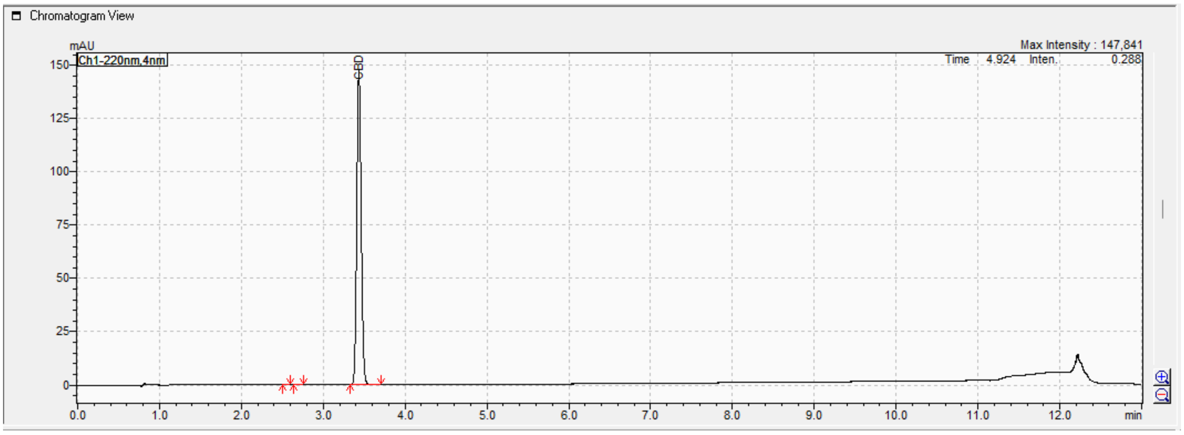

Figure S5. – HPLC chromatogram of pure Cannabidiol (CBD).

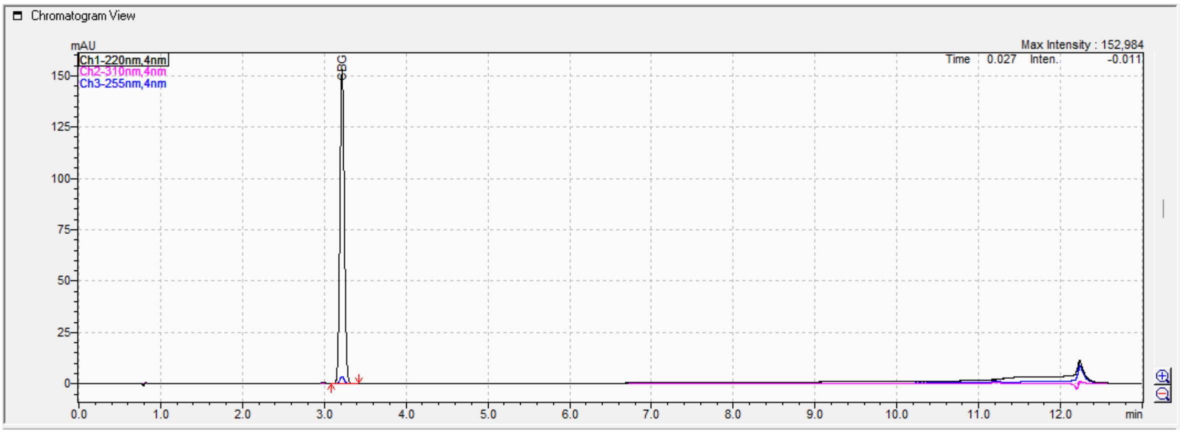

Figure S6. – HPLC chromatogram of pure Cannabigerol (CBG).

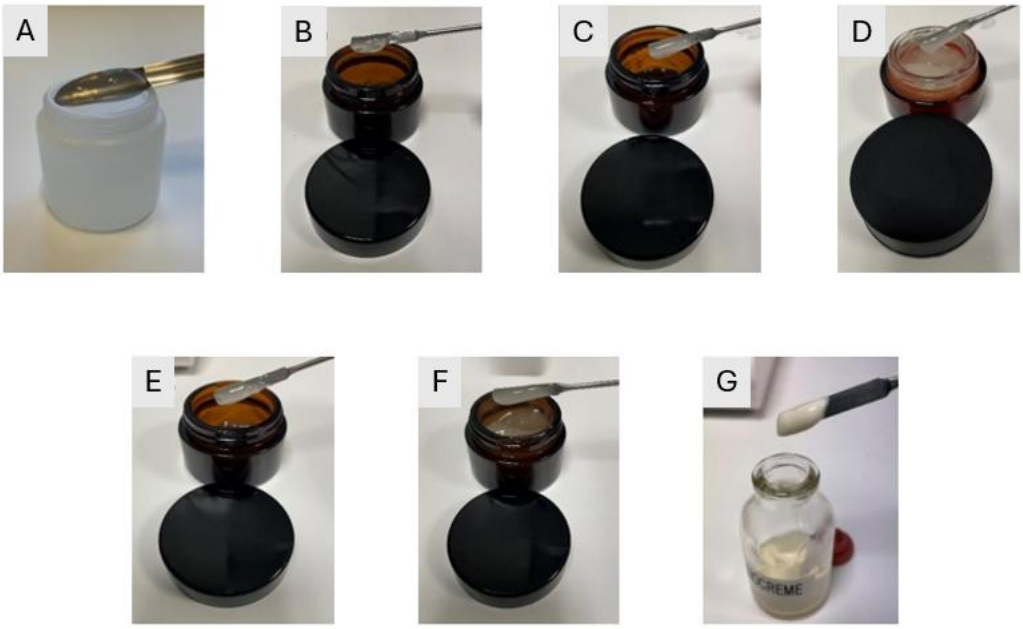

Figure S7. –Appearance of loaded and unloaded formulations based on gel A) Placebo Carbopol® 940 gel and containing B) mixture of cannabinoids (Cann); C); essential oil of FB; D) FB.Cann; E) essential oil of FC; F) FC.Cann; G) Positive Control 2 – commercial cream containing a cannabinoid.

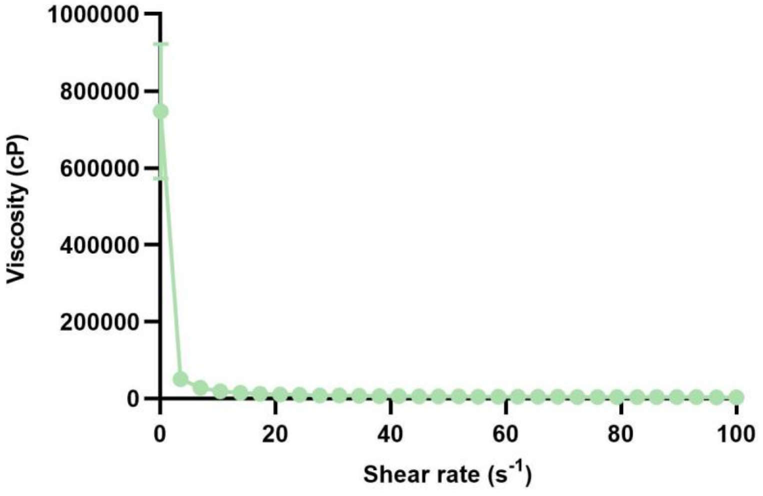

Figure S8. – Measurement of viscosity (cP) against the shear rate (s-1) of Placebo Carbopol® 940 gel using the rheometer.

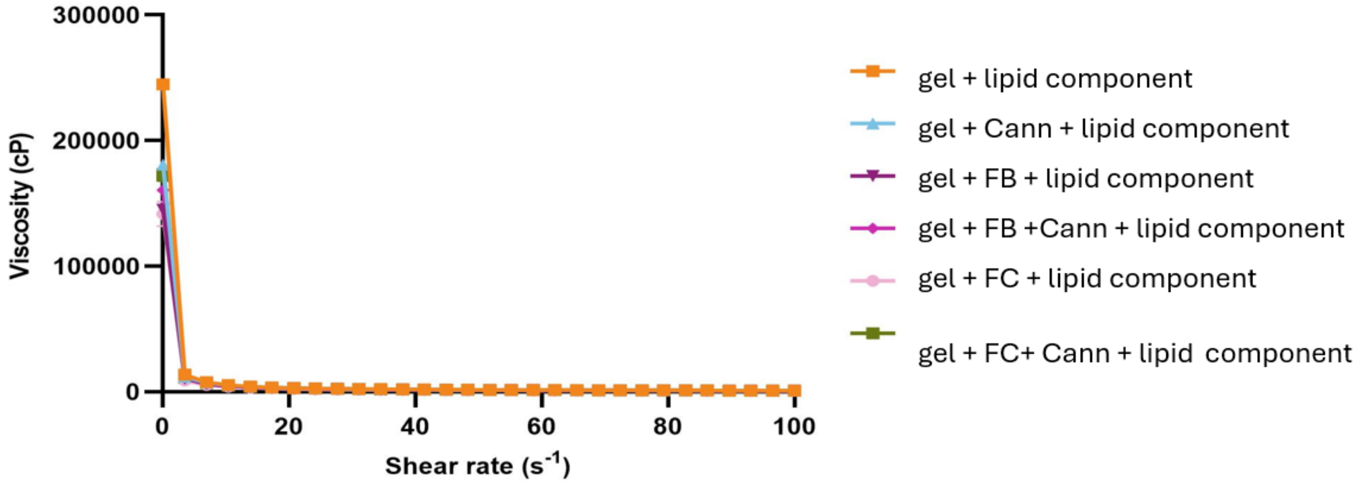

Figure S9 – Measurement of viscosity (cP) against shear rate (s^{-1}) of the gel + 1 % (*w/w*) of lipid component (n=1); gel + mixture of cannabinoids (Cann) + 1 % (*w/w*) of lipid component (n=1); gel + 5 % (*w/w*) of FB + 1 % (*w/w*) of lipid component (n=1); gel + 5 % (*w/w*) of FB + mixture of cannabinoids (Cann) + 1 % (*w/w*) of lipid component (n=1); gel + 5 % (*w/w*) of FC + 1 % (*w/w*) of lipid component (n=2); gel + 5 % (*w/w*) of FC + mixture of cannabinoids (Cann) + 1 % (*w/w*) of lipid component (n=3), using the rheometer.

Table S1. – Major terpenes present in fractions of interest, FA, FB and FC and the respective Relative Retention Indices (RRI) determined by GC-MS.

Fraction of interest	Major terpenes	RRI
**FA**	Thymol	1262
	Linalool	1082
**FB**	Eucalyptol	1059
	α-Terpineol	1143
**FC**	γ-Terpinene	998
	Terpinolene	1052

Table S2. Analysis of the loaded and unloaded formulations based on gel: Placebo Carbopol® 940 gel, Cann – gel + mixture of cannabinoids, FB – gel + FB; FB.Cann – gel + FB + mixture of cannabinoids; FC – gel + FC; FC.Cann – gel + FC + mixture of cannabinoids and Positive Control 2 – commercial cream containing a cannabinoid.

	**Placebo Carbopol® gel**	**Cann**	**FB**	**FB.Cann**	**FC**	**FC.Cann**	**Positive Control 2**
**Appearance***	Colorless	Translucent		Opaque			White
**Homogeneity**	Homogeneous						
**Texture**	Light weight						Thick
**Odour**	Odourless	Odour					
**Adhesion**	Robust and adherent to the skin						

Table S3. Preliminary Stability Testing carried out at 25 ºC and - 5 ºC (corresponding to the heating and cooling assay) and under centrifuge stress, with Placebo Carbopol® 940 gel, Placebo Carbopol® 940 gel + FB + mixture of cannabinoids (FB.Cann) and Placebo Carbopol® 940 gel + FC + mixture of cannabinoids (FC.Cann). The analysis was carried out through organoleptic characterization (OC), pH and percentage variation in terms of viscosity compared to day 0 and before being subjected to centrifugation. Data is represented as the average of the experiments carried out on each test day, per formulation (n=2).

**Placebo Carbopol® gel**				**Carbopol® gel + FB.Cann**				**Carbopol® gel + FC.Cann**			
**Preliminary Stability Testing (Heating and Cooling)**											
Days	OC	pH	Variation in viscosity vs time zero (%)	Days	OC	pH	Variation in viscosity vs time zero (%)	Days	OC	pH	Variation in viscosity vs time zero (%)
0	N	5 – 6	-	0	N	5 – 6	-	0	N	5 – 6	-
2	N	5 – 6	2.42	2	N	5 – 6	-13.93	2	N	5 – 6	-1.58
4	N	5 – 6	-3.08	4	N	5 – 6	-11.77	4	N	5 – 6	-16.85
7	N	5 – 6	0.50	7	N	5 – 6	-8.89	7	N	5 – 6	-9.26
**Preliminary Stability Testing (Centrifugation Stress)**											
**Before**	N	5 – 6	-	**Before**	N	5 – 6	-	**Before**	N	5 – 6	-
**After**	N	5 – 6	-7.20	**After**	N	5 – 6	4.26	**After**	N	5 – 6	-8.69

N: normal physical appearance
